# First imported cases of MPXV Clade Ib from Goma, Democratic Republic of the Congo

**DOI:** 10.1038/s43856-025-01203-z

**Published:** 2025-11-15

**Authors:** Daniel Mukadi-Bamuleka, Eddy Kinganda-Lusamaki, Noella Mulopo-Mukanya, Adrienne Amuri-Aziza, Áine O’Toole, Sydney Merritt, Brigitte Modadra-Madakpa, Guy Mutombo-Ndongala, Emmanuel Hasivirwe Vakaniaki, Cris Kacita, Gaston Lubambo Maboko, Jean-Claude Makangara-Cigolo, Michel Ngimba, Emmanuel Lokilo-Lofiko, Elisabeth Pukuta-Simbu, Gradi Luakanda-Ndelemo, Tavia Bodisa-Matamu, Zephanie Paluku-Kalimuli, Prince Akil-Bandali, Sifa Kavira-Bapitani, Daan Jansen, Adèle Kavira-Kamaliro, Emile Muhindo-Milonde, Jeriel Mufungizi, Yves Birindwa-Hamisi, Hugo Kavunga, Olivier Tshiani, Sabin Sabiti Nundu, Laurens Liesenborghs, Nicole A. Hoff, Megan Halbrook, Robert Shongo, Ahidjo Ayouba, Genay Pilarowski, Alain Kakule Mangolopa, Amos Kiuka Ebondo, Nicola Low, Souradet Y. Shaw, Sam Wilkinson, Sofonias Kifle Tessema, Lorenzo Subissi, Eric Delaporte, Koen Vercauteren, Tony Wawina-Bokalanga, Anne W. Rimoin, Lisa E. Hensley, Martine Peeters, Nicholas Loman, Andrew Rambaut, Jean-Jacques Muyembe-Tamfum, Jason Kindrachuk, Placide Mbala-Kingebeni, Steve Ahuka-Mundeke

**Affiliations:** 1 Service de Microbiologie, Département de Biologie Médicale, Cliniques Universitaires de Kinshasa, Université de Kinshasa, Kinshasa, Democratic Republic of the Congo; 2 Rodolphe Merieux INRB-Goma Laboratory, Goma, Democratic Republic of the Congo; 3 Institut National de Recherche Biomédicale (INRB), Kinshasa, Democratic Republic of the Congo; 4 TransVIHMI (Recherches Translationnelles sur le VIH et les Maladies Infectieuses), Université de Montpellier, Institut de Recherche pour le Développement (IRD), Institut National de Santé et de Recherche Médicale (INSERM), Montpellier, France; 5 Institute of Ecology and Evolution, University of Edinburgh, Edinburgh, UK; 6 Department of Epidemiology, Jonathan and Karin Fielding School of Public Health, University of California, Los Angeles, USA; 7 Ministry of Public Health, Hygiene and Social Welfare; Provincial Health Division, Goma, North Kivu Democratic Republic of the Congo; 8 Hemorrhagic Fever and Monkeypox Program, Ministry of Health, Kinshasa, Democratic Republic of the Congo; 9 Graduate School of Cellular and Biomedical Sciences, University of Bern, Bern, Switzerland; 10 Department of Clinical Sciences, Institute of Tropical Medicine, Antwerp, Belgium; 11 Department of Microbiology, Immunology and Transplantation, KU Leuven, Leuven, Belgium; 12 Chan Zuckerberg Biohub, San Francisco, USA; 13 World Health Organization, DRC country Office, Kinshasa, Democratic Republic of the Congo; 14 Institute of Social and Preventive Medicine, University of Bern, Bern, Switzerland; 15 College of Community and Global Health, University of Manitoba, Winnipeg, Canada; 16 Department of Medical Microbiology & Infectious Diseases, University of Manitoba, Winnipeg, Canada; 17 University of Birmingham, Birmingham, UK; 18 Africa Centres for Disease Control and Prevention, Addis Ababa, Ethiopia; 19 World Health Organization, Geneva, Switzerland; 20 US Department of Agriculture, Agricultural Research Service, Manhattan, USA; 21 Department of Internal Medicine, University of Manitoba, Manitoba, Canada; 22 South African National Bioinformatics Institute, University of the Western Cape, Cape Town, South Africa; 23 Osaka Metropolitan University, Osaka, Japan

**Keywords:** Epidemiology, Pox virus

## Abstract

**Background:**

The ongoing mpox outbreaks in the Democratic Republic of the Congo (DRC) resulted in >71,000 suspected cases from 01 January 2024 to 02 February 2025. Clade Ib mpox virus (MPXV) emergence has heightened public health concern due to observed sustained human-human transmission and spread to multiple non-endemic East African countries. Clade Ib outbreaks have been marked by epidemiologic deviations from classic Clade Ia zoonotic transmission—Clade Ib instead has been observed among adult populations and transmission via sexual contact. With the continued expansion of Clade Ib across the region, containment and mitigation measures may need to be adapted to best fit this novel MPXV clade.

**Methods:**

Case investigation and epidemiological assessment data as well as whole viral geonome sequencing was analyzed from confirmed mpox infected individuals in the Goma region. Case demographics and clinical presentation data was also assessed from suspected mpox cases in the region.

**Results:**

We report the first introduction of Clade Ib into North Kivu province through close contact transmission. We also report limited human-human Clade Ib transmission chains among children <15 years in the Mudja internal displaced persons camp. We further present evidence of APOBEC3 mutations and genomic links between these North Kivu cases with the larger ongoing Clade Ib outbreak in Kamituga, South Kivu.

**Conclusions:**

Given the expansion of regional mpox outbreaks and populations considered at-risk, these findings underscore how mpox case investigations and community messaging should include considerations for non-sexual human-human transmission of Clade Ib that includes children <15 years.

## Plain language summary

Mpox virus, which is endemic in tropical, forested regions of Central and West Africa, has resulted in mpox outbreaks in the Democratic Republic of the Congo (DRC) with historic infections, including more than 71,000 suspected cases reported between January 2024 and February 2025. In recent years, shifting clinical and epidemiological characteristics have been reported for Clade I Mpox virus (MPXV), which is endemic in DRC. This includes the emergence of a new subclade, Clade Ib MPXV, first identified in South Kivu, DRC, in 2023. We report the first introduction of Clade Ib into North Kivu province through close contact transmission. This includes limited human-human transmission of MPXV among children in an internally displaced persons camp, raising concerns regarding undetected transmissions among these vulnerable groups. We further present evidence for links between these North Kivu cases with the larger ongoing outbreak in Kamituga, South Kivu. These findings underscore how mpox case investigations and community messaging should include considerations for non-sexual human-human transmission of Clade Ib that includes children and vulnerable populations.

## Abstract (web summary)

Mukadi-Bamuleka et al., report the first introduction of MPXV Clade Ib into Goma, DRC, revealing non-sexual human-to-human transmission chains involving children in an internally displaced persons’ camp. These findings underscore the urgent need to expand surveillance and public health messaging beyond adult sexual networks to include paediatric and displaced populations.

## Introduction

Mpox is an emerging zoonotic disease caused by the mpox virus (MPXV). Following the global 2022 mpox outbreak marked by MPXV expansion via broad human-human transmission, the disease has drawn significant international attention. This multi-national outbreak was concentrated within dense sexual networks in more than 100 historically non-endemic countries^1^, resulting in a first declaration of a public health emergency of international concern (PHEIC) by the World Health Organization^2–4^. Mpox was first described in 1970 in the Democratic Republic of the Congo (DRC). Since the disease has been considered endemic among tropical forested regions of Central and West Africa^5^. While over the past five decades, mpox outbreaks have been sporadically reported, there has been a generally increasing burden of disease across endemic areas, with the DRC facing the greatest public health impact^2,6,7^. This increasing burden has coincided with the parallel decrease in population immunity to orthopoxviruses. Indeed, the cessation of the global smallpox vaccination program has increased the number of immune-naive individuals over time^7–9^. Recently, a surge in cases in the African region and the emergence of a new MPXV subclade, Ib, prompted the second declaration of a PHEIC in August 2024^10^.

In endemic regions, historical mpox cases were primarily associated with zoonotic transmission, followed by limited secondary cases among close contacts. However, sustained human-to-human transmission was noted with mpox re-emergence in Nigeria in 2017 and the 2022 global mpox outbreak^11,12^. Close sexual (intimate) contact and altered or atypical clinical disease presentation were highly overrepresented among cases during the global outbreak^3,13^, with 96% of cases reported in males, among which 87% self-identified as men who have sex with men^14^.

Since early 2023, sustained human-to-human transmission and expansion of mpox have been described across DRC, with sexual contact-associated transmission reported for the first time in Kwango and South Kivu provinces^15–18^. This has extended to all DRC provinces and many large cities, including those with no historical reported mpox cases. Eastern DRC has faced multiple decades of political unrest, resulting in population displacement and the establishment of internally displaced persons (IDP) camps, hunger, and a deteriorated health infrastructure. However, the region is also known to have high population mobility, good road infrastructure, internal and international trade, and established mining activities^19^.

MPXV Clade Ib first emerged in Kamituga Health Zone (HZ), South Kivu province, in September 2023. Among the first clusters of confirmed cases, 51.9% were women, including 29.0% self-reported sex workers, and the median age was 22 years^15^. This was a marked departure from the 2022 global outbreak of Clade IIb mpox, where infections were predominantly identified among males. Genomic sequencing of MPXV from Kamituga samples revealed APOBEC3-like mutations in high-quality complete genomes, prompting the subdivision of MPXV Clade I into subclades Ia and Ib. The latter was linked to sustained human-to-human transmission trends^15^, whereas the former was predominantly associated with zoonotic transmission^20^. Molecular clock analysis suggested that Clade Ib MPXV has been circulating in Kamituga since mid-September 2023 (95% highest posterior density intervals July 2023–October 2023)^15^. Here, we describe the introduction of MPXV Clade Ib to North Kivu province, including the Mudja IDP camp, and limited human-human transmission among children.

## Methods

### Ethical considerations

This study was exempted from ethical approval since it was conducted as part of the national surveillance activities under the authorization of the DRC Ministry of Health. However, permission was granted by the Kinshasa School of Public Health Ethics Committee to use anonymized data from the surveillance and laboratory (ESP-UNIKIN, Number ESP/CE/05/2023). De-identified data were provided by the National Programme for Mpox and Viral Hemorrhagic Fevers Control (PNLMPX-VHF) and the National Institute for Biomedical Research (INRB), both organizations under the DRC National Institutes of Public Health.

### Suspected mpox case investigations

Human mpox is a mandatory reportable disease in the DRC. The official case definition was established by the Ministry of Health in 2001 and amended in 2010 to enhance surveillance of mpox in Tshuapa Province. The case definition for suspected mpox reads as follows: (1) a patient with a vesicular pustular eruption (hard and deep pustules), and (2) at least one of the following symptoms: fever preceding the eruption, lymphadenopathy (inguinal, axillary, or cervical), and/or pustules or crusts on the palms of the hands or soles of the feet, and exposure history (travel from an affected area, a high risk contact with people coming from affected area, or exposure to wild animal dead or presenting lesions)^21^. Formal investigation of a suspected mpox case includes the collection of samples and the completion of a case investigation form by trained staff from HZ, where the case was reported, and the Provincial Health Division.

### Sample collection and laboratory diagnosis of mpox

Lesion swabs and/or crusts are preferred samples for laboratory diagnosis, although blood and nasopharyngeal secretions can also be collected. Specimens collected were shipped to the Rodolphe Merieux INRB-Goma Laboratory for qPCR assessment and whole genome sequencing (WGS). MPXV was confirmed by qPCR on the Radi^®^ platform (KH Medical, Seoul, South Korea) according to the manufacturer’s instructions. Mpox positive samples with cycle threshold (Ct) values < 30 were selected for subsequent WGS using amplicon-based enrichment on both Illumina and ONT platforms^22^.

### Bioinformatics analysis

FASTQ files from GridION X5^®^ (Oxford Nanopore Technologies, Oxford, UK) were base-called with the high-accuracy model from Guppy v6, and reads were demultiplexed and adapter-trimmed by the GridION built-in MinKNOW^®^ software. MPXV consensus genomes were generated using the artic (https://github.com/artic-network/artic-mpxv-nf) and metatropics pipelines (https://github.com/DaanJansen94/nextflow-metatropics-INRB). Fastq files from Iseq^TM^100 (Illumina) were analyzed using CZid for consensus genome generation. Genomes with coverage above 80% were selected for phylogenetic analysis.

### Phylogenetic and APOBEC3 analysis

We estimated a maximum likelihood phylogeny using IQ-TREE 2 version 2.2.5^23^ with the Hasegawa, Kishino, Yano (HKY) substitution model. Ancestral reconstruction was performed for each internal node on the phylogeny using IQ-TREE 2, enabling mapping of single-nucleotide polymorphisms (SNPs) along branches. SNPs were categorized based on whether they were consistent with the signature of APOBEC3 editing, assuming this process induced specific mutations (TC  →  TT and GA  →  AA) as previously described^24,25^. Further phylogeographic investigation was performed using the Nextstrain pipeline to integrate other clade Ib genomes detected in South Kivu, in other provinces and countries^26^.

### Reporting summary

Further information on research design is available in the Nature Portfolio Reporting Summary linked to this article.

## Results

### Case investigation and epidemiological assessment

**Fig. 1:**
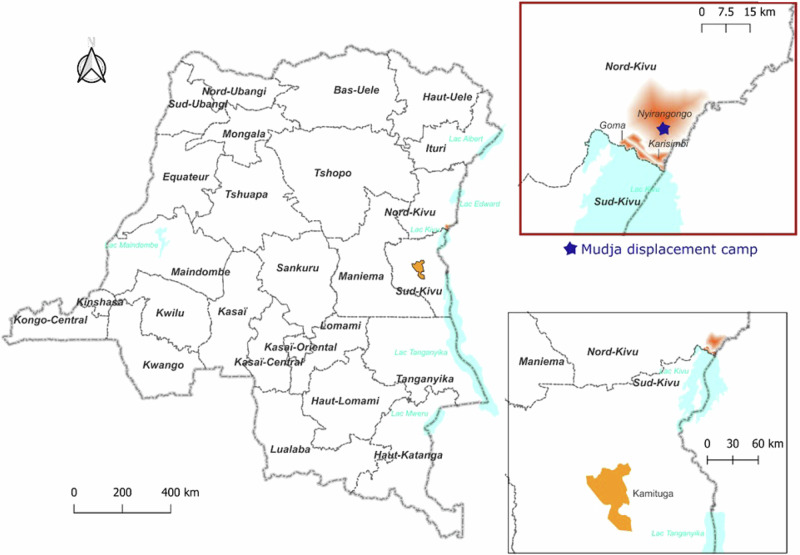
Geographic locations of cases identified in North Kivu, including the Mudja displacement camp. Map constructed using QGIS3.22.11. The fading red coloring on the inset signifies the three Goma health zones where the sequenced samples originated from. The star representing the Mudja (or Muja) camp location within the Nyiragongo health zone, utilizing coordinates from Google maps and OpenStreetMap.

**Fig. 2:**
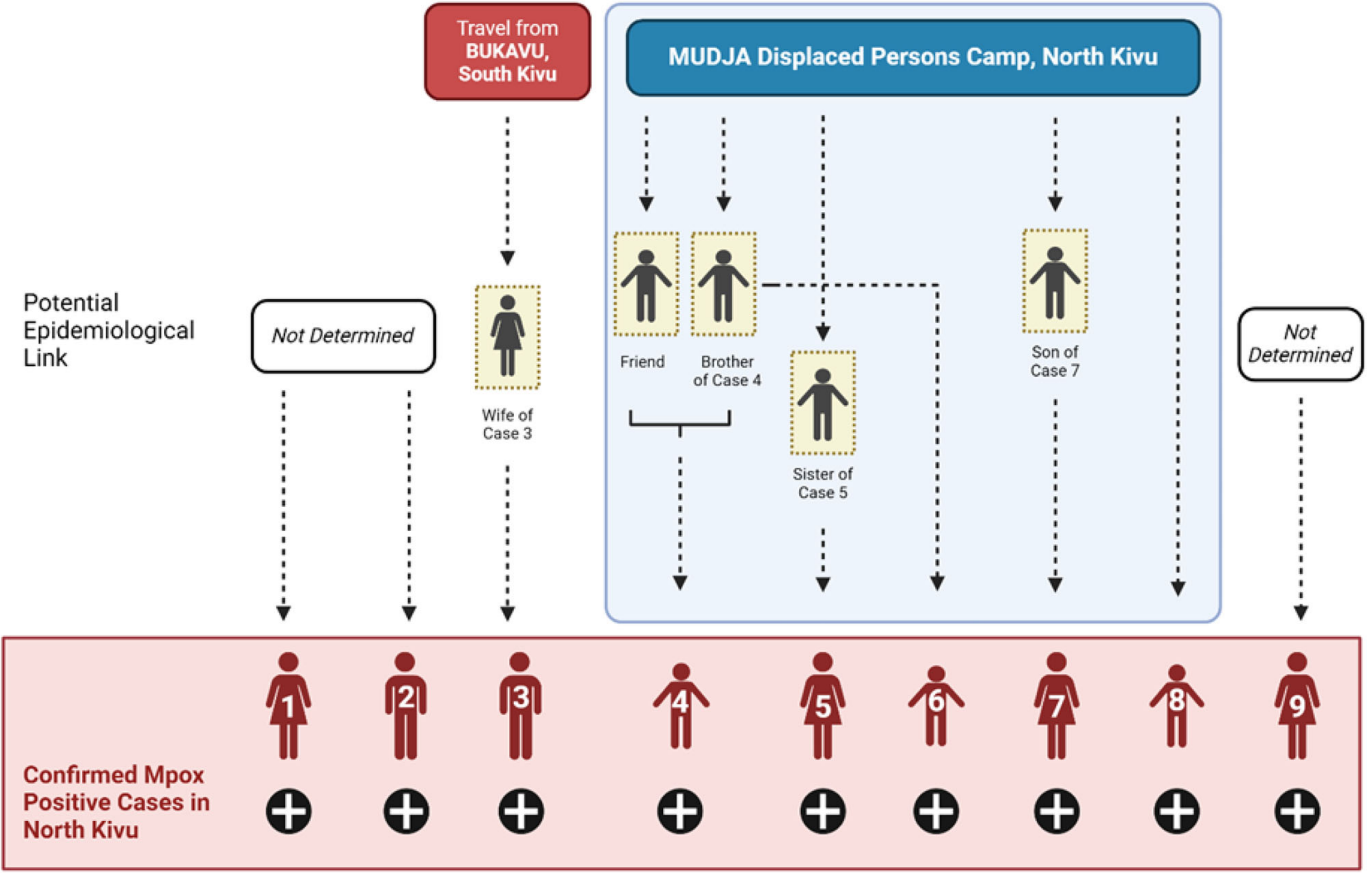
Epidemiological linkages and suspected transmission chains among Clade Ib mpox cases identified in Goma, North Kivu, DRC. Transmission chain at the Division Provincial de la Santé (DPS), North Kivu. Clade Ib MPXV has been confirmed for Cases 1–3, and results are pending for cases 4–9 at this time.

We describe a total of nine mpox cases, of which six were sequenced and confirmed as MPXV Clade Ib. A map of the geographic locations of the identified cases is presented in Fig. 1. Epidemiological links among confirmed Cases 1–9 are presented in Fig. 2.

Confirmed Case 1, an adult female (15–30 years) from North Kivu, developed vesicular and pustular lesions on day 5 post-symptom onset, and healthcare consultation in May 2024. She was discharged 1 day later (day 6) and cared for at home. Vesicle and nasopharyngeal swabs were collected on day 8 post-symptom onset and confirmed positive for orthopoxvirus by qPCR. Syphilis rapid plasma reagin (RPR) and HIV (Determine^®^) tests were negative. She reported travel to South Kivu 21 days prior to symptom development, although this could not be confirmed. Subsequent investigations of contacts at the healthcare facility (*n* = 23) and their residences (*n* = 19) did not identify additional mpox cases.

Confirmed Case 2, an adult male (15–30 years) from North Kivu, consulted medical care for vesicular lesions in June 2024. Lesions were swabbed and laboratory-confirmed MPXV-positive the day following the consultation. Upon further investigation, Case 2 reported that he had had sexual intercourse with a casual partner 21 days prior to symptom onset, though he did not notice any visible lesions on his or his partner’s body.

Confirmed Case 3, an adult male (15–30 years) from North Kivu, consulted medical care 8 days post-symptom onset and tested positive for MPXV by qPCR the following day. He reported that his spouse had consulted a healthcare professional for similar symptoms a few days ago. Following investigation, an epidemiological link was established between confirmed Case 3 and his spouse, who had reported a recent history of travel to South Kivu 21 days prior to symptom onset (probable Case 1). No epidemiological link was established between confirmed Cases 1–3.

Five additional confirmed mpox cases (Cases 4–8) were residents of the Mudja IDP camp, North Kivu. Probable epidemiological links were made among Cases 4–6 based on frequent interactions among residents within the camp, including probable mpox cases.

Confirmed Case 4, a male child (<15 years), developed fever and headache. The child reported shared use of a sponge that had been used on his male sibling (<15 years), who recently had dermatological lesions like Case 4, though no confirmatory testing had been performed (probable Case 2) at that time. The sibling of Case 4 had also interacted with a neighbor who had developed skin lesions a few days prior to this probable Case 2. However, there was no epidemiological or testing information available (probable Case 3). Of note, Case 4 and his male sibling shared sleeping quarters. Skin eruptions, including vesicles and pustules, were noted for Case 4 on day 3 post-symptom onset, and qPCR confirmed MPXV with samples collected on day 5.

Confirmed Case 5, an adult female (15–30 years), developed symptoms in mid-June 2024, including chills, cough, headache, lymphadenopathy, eruptions on the lips, and pubic lesions. A female child living within the same household had reportedly developed similar symptoms ~14 days earlier but had not sought clinical care (probable Case 4).

Confirmed Case 6, a male child (<15 years), presented lesions on the back, face, and abdomen. Case 6 reported being a close contact (friend) of probable Case 2.

Confirmed Case 7, an adult female (>30 years), developed a rash on the buttocks, thighs, hands, and feet. No epidemiological link with other cases in the Mudja camp was established with Case 7. However, she reported having provided recent care for her son (<15 years), who presented a similar illness and with whom she shared the bed (probable Case 5).

Confirmed Case 8, a female child (<15 years) living in Mudja camp, presented with fever on the day of symptom onset, followed by vesicular eruptions on the thighs, abdomen, back, and face, without adenopathies. An epidemiological link has not been clearly established with other probable or confirmed cases in the camp; despite reporting she had been in contact with other children in Mudja.

Confirmed Case 9, an adult female (15–30 years), presented with vesiculo-pustular lesions on day 3 post-symptom onset (fever starting on day 0). Samples were tested positive for MPXV on day 10. No epidemiological link was established with any other cases.

### Case demographics and clinical presentation

Of all confirmed cases, five (5/9) were residents of the Mudja IDP camp, with 5 males and 4 females. The mean age was 18 years (6–45 years). Most cases were identified in the 15–30 age range (5/9), and three were aged <15 years. Most patients (6/9) required hospitalization, including three males and three females; 3/6 were aged 15–30 years, 2/6 were <15 years, and 1/6 was >30 years. No fatalities were recorded among all confirmed cases. Skin lesions were reported in all patients (9/9), with frequent genital and oral eruptions (7/9 and 6/9 cases, respectively). Genital eruptions were similarly reported in males (4/5) and females (3/4). Oral lesions were mostly reported among females (4/4) than males (2/4). Myalgia (8/9), headache (7/9), and fever (6/9) were most common among mpox patients. Lymphadenopathy was frequent with the following locations: cervical (7/9), inguinal (5/9), and axillary (3/9).

As of October 8, 2024, 170 suspect cases were investigated in North Kivu province (Table 1). They presented similar clinical symptoms to the investigated cases, with 92.4% with cutaneous lesions and 82.4% with fever. Additionally, the demographic composition of this larger suspect mpox cohort was similar to the nine confirmed cases—55.6% of cases were male, with the majority of suspect cases aged 15–30 years old. Furthermore, the hospitalization rate of the 170 suspect cases—60.6%—was comparable to the nine PCR-confirmed cases.

**Table 1:** Demographic and clinical symptom data for confirmed mpox cases (*n* = 9) and suspected mpox (*n* = 170) identified in the Goma region to date

Characteristic	Confirmed Mpox Cases (*n* = 9)		Suspect Mpox cases (*n* = 170)	
	*N*	%	*N*	%
Reported health zone				
Goma HZ	0	0.0	114	67.1
Karisimbi HZ	0	0.0	42	24.7
Nyiragongo HZ	5	55.6	0	0.0
Not specified	4	44.4	14	8.2
Sex				
Male	5	55.6	94	55.3
Female	4	44.4	75	44.1
Not reported	0	0.0	1	0.6
Age group				
<15	3	33.3	48	28.2
15–30	5	55.6	87	51.2
>30	1	11.1	33	19.4
Clinical symptoms				
Cutaneous eruptions	9	100.0	157	92.4
Genital eruptions	7	77.8	45	26.5
Oral eruptions	6	66.7	17	10.0
Fever	6	66.7	140	82.4
Headache	7	77.8	78	45.9
Myalgia	8	88.9	52	30.6
Arthralgia	7	77.8	43	25.3
Fatigue	3	33.3	60	35.3
Cervical lymphadenopathy	7	77.8	40	23.5
Inguinal lymphadenopathy	5	55.6	38	22.4
Axillary lymphadenopathy	3	33.3	31	18.2
Hospitalization	6	66.7	103	60.6

Column percentages are presented in parentheses.

### Genome sequencing

**Fig. 3:**
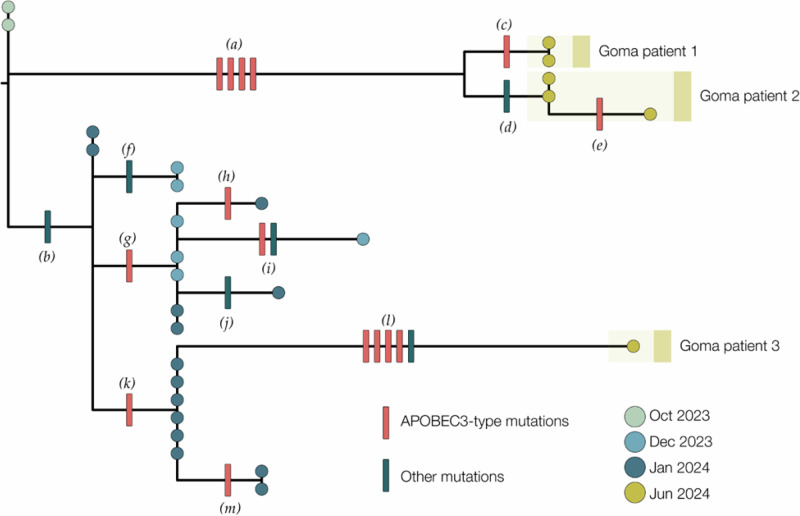
A maximum likelihood tree constructed using IQ-Tree 2^23^ with the HKY substitution model^34^. We included a Clade Ia genome as an outgroup and then removed it after rooting. Single-nucleotide mutations are reconstructed and displayed, denoting whether they are APOBEC3-mediated (red bars) or other mutations (blue bars). Genomes from recent cases in Goma, North Kivu, are denoted with yellow circles. Patients 1 and 2 each have multiple genomes sequenced from different samples. Blue circles are genomes from Kamituga, South Kivu, from October and December 2023, and January 2024. Letters in parentheses are referenced in Table 2.

Genomic analysis of the first three confirmed cases suggests that all cases clustered within MPXV Clade Ib, much like mpox cases detected in South Kivu (Fig. 3). Their position in the tree with MPXV sequences from South Kivu suggests they were part of the sustained human outbreak first reported in Kamituga HZ. These findings are consistent with travel history to South Kivu as reported by the first case. Genomes for Cases 1 and 2 are closely linked, suggesting that they were part of the same transmission chain, albeit no epidemiological link was established through investigation. The third sequence is separated from Cases 1 and 2 in the phylogenetic tree, implying an independent introduction into Goma.

Of the 21 single-nucleotide mutations reconstructed in the phylogenetic tree (Table 2), 15 were expected due to the action of human APOBEC3. The viruses sequenced from Cases 1 and 2 descended from viruses circulating in Kamituga in October 2023, or earlier. Furthermore, the branch leading to Cases 1 and 2 has four APOBEC3-type mutations, which, even at the elevated rate of evolution induced by APOBEC3, would represent months of human transmission. In combination with the reported recent travel history, sequence data indicate a likely recent introduction into the Goma area from the South Kivu region.

**Table 2:** List of mutations in the 2023-2024 DRC mpox outbreak

Branch label^a^	Genome position^b^	Mutation^c^	APOBEC3?
*(a)*	103,660	GA->AA	yes
	130,155	GA->AA	yes
	142,587	GA->AA	yes
	161,978	TC->TT	yes
*(b)*	165,597	C->A	no
*(c)*	64,117	GA->AA	yes
*(d)*	30,660	T->C	no
*(e)*	181,268	GA->AA	yes
*(f)*	167,805	A->G	no
*(g)*	115,484	GA->AA	yes
*(h)*	57,332	GA->AA	yes
*(i)*	89,000	GA->AA	yes
	127,619	A->G	no
*(k)*	183,181	G->A	no
*(l)*	71,751	TC->TT	yes
	139,485	TC->TT	yes
	148,221	GA->AA	yes
	168,749	GA->AA	yes
	169,501	T->A	no

^a^Branch labels refer to Fig. 3.

^b^Coordinates relative to Clade I reference genome, NCBI accession NC_003310.

^c^Dinucleotide mutations are ascribed to APOBEC3 mutations.

**Fig. 4:**
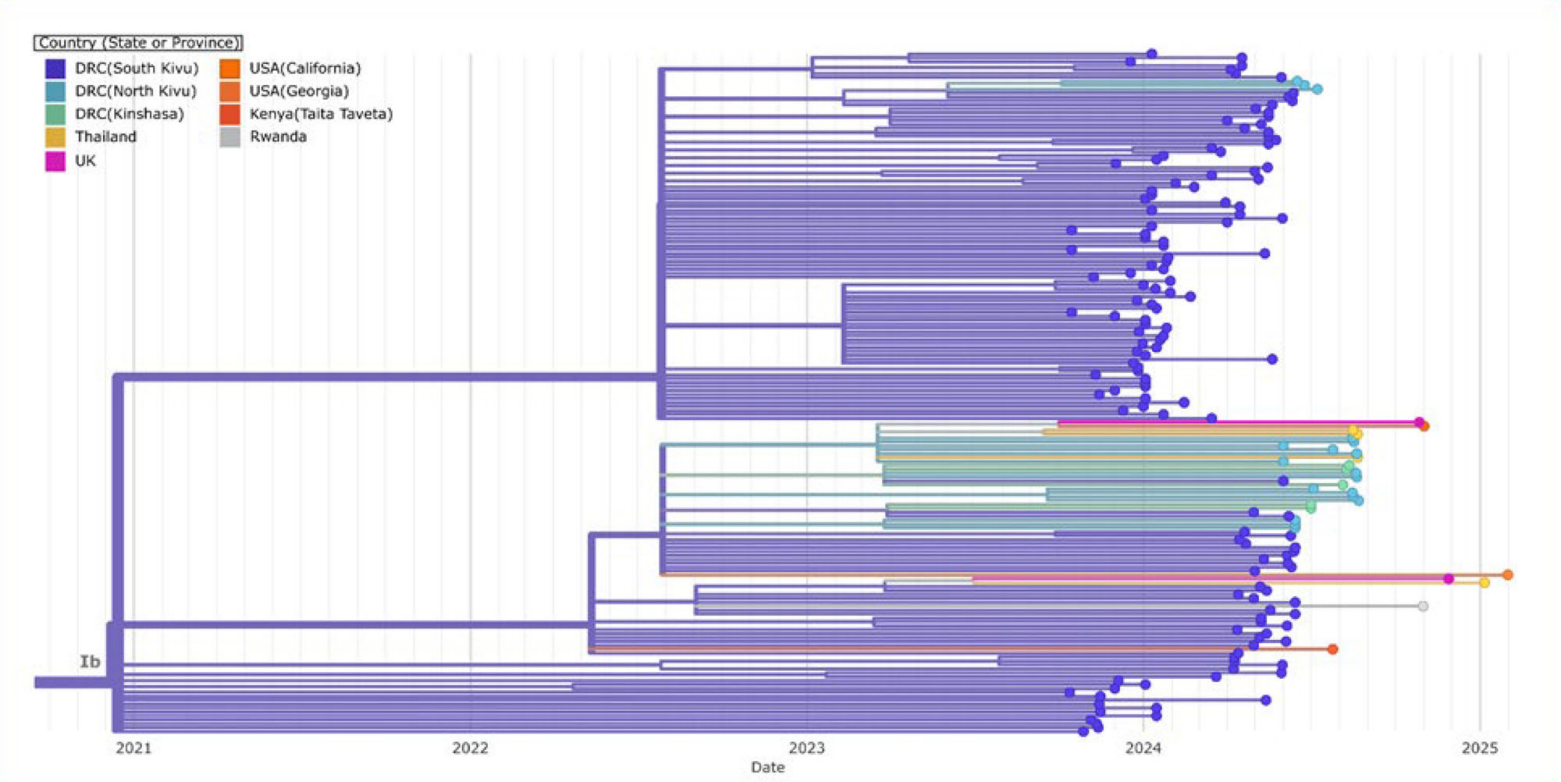
Clade Ib Timetree. The Clade Ib Timetree was built with https://github.com/nextstrain/mpox, showing 174 genomes, including 18 from North Kivu in moderate cyan (6 from the first cases described in Fig. 3 and 12 generated later), 141 from South Kivu in moderate blue, 5 from Kinshasa in lime green, and 10 from other countries in other colors. To note that this is a subtree, and the overall tree has been built with 598 genomes.

**Fig. 5:**
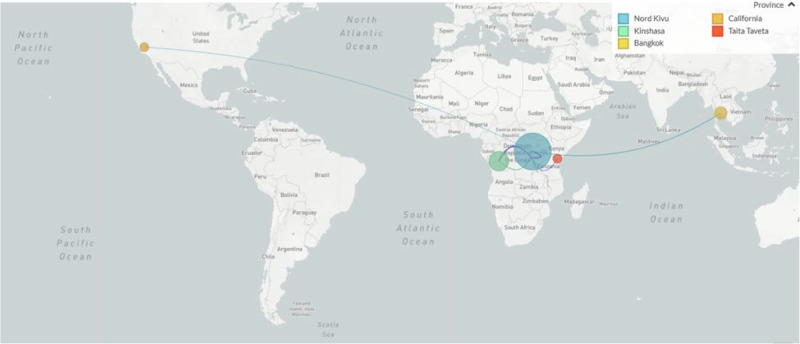
Phylogeographic relationship between clade Ib genomes. To note, South Kivu genomes have been filtered out for visualization purpose.

The branch leading to Case 3 has four APOBEC3 mutations and one other mutation, possibly the result of an error during replication, indicating a similar timespan for this branch. However, this branch joins the Kamituga tree clustering with viruses sampled in January 2024, suggesting a direct link to that outbreak. The long unbroken branch with five single-nucleotide mutations, and the fact that this lineage has not yet been sampled in Kamituga, may either be explained by under-sampling in Kamituga, or an unresolved epidemiological link to South Kivu (i.e., by not having identified or sequenced the contact of Case 3, who may also be epidemiologically linked to South Kivu). However, the third hypothesis, being that the virus has been circulating in Goma for weeks or months, cannot be entirely excluded. Further investigation, including 11 additional genomes from samples collected in July and August 2024, showed that the genomes from North Kivu are in the same clusters as some of the viruses detected outside of Africa, including Thailand, the UK, and the USA (California) (Figs. 4 and 5). All accession or labID designations are provided in Supplementary Table 1.

## Discussion

Here, we present, to the best of our knowledge, the first case series of Clade Ib mpox reported in North Kivu province, DRC, with probable introduction to Mudja IDP camp. Case investigations and epidemiological analysis demonstrate non-sexual contact transmission of Clade Ib MPXV, especially among children. The investigation also suggests a potential linkage of cases to recent travel history to South Kivu (a hotspot of the Clade Ib mpox outbreak). The introduction and expansion of mpox in large urban centers of DRC, including Bukavu (>1 million inhabitants) and Goma (~2 million inhabitants), further increases the risk for expanded public health impacts in the region. Of note, we have identified an MPXV Clade Ib variant circulating within large cities (Goma, Bukavu, Kamituga, and Kinshasa) and continued expansion of the subclade through sustained human-to-human transmission^15,17,18,27–29^. This risk is compounded by the role of Goma as a major commercial and air travel hub^30,31^. Additionally, the proximity of Goma to international borders (Uganda, Rwanda, Burundi, and Tanzania) and frequent unmonitored cross-border movement increases the potential for the virus to spread to new regions through cryptic transmission^30^.

A concerning finding from this investigation was the identification of mpox cases among individuals in the Mudja IDP camp. The introduction of MPXV to, and circulation within, an IDP camp could have deleterious public health impacts. Importantly, this includes the potential for broad disease transmission facilitated by poor sanitation conditions, highly dense populations, and very limited healthcare or surveillance support. Additionally, ongoing political unrest in the region could further destabilize mpox containment and mitigation efforts.

The investigations among the nine confirmed mpox cases in this study have identified potential household contacts with unreported symptomatic infections. This highlights the need for greater community engagement focused on case recognition and suspected case reporting. Additionally, the case investigations also noted the potential for contact-mediated transmission among children through common play activities. Given the cases identified herein among children and considering the disproportionate disease severity associated with mpox among children, additional vigilance will be required to inform communities of potential risks for mpox spread within children. Of important note was the potential linkage of Case 1 to recent travel to Bukavu, South Kivu. Ongoing conflict within the region could also impact the containment and mitigation efforts for MPXV circulation within Mudja, given the potential for onward transmission among residents as well as further introductions of the virus through undiagnosed infections. Assessment of Clade Ib introduction and circulation in Mudja by viral genome sequencing is ongoing from samples collected during this investigation.

Our investigation also demonstrated that risks of infection for recently identified MPXV Clade Ib extend beyond adult sexual (intimate) contacts, including other adults, caregivers, and children. These observations highlight the increasingly complex epidemiology of mpox and the potential for broader public health impacts that are context-dependent. This should be considered with the caveat of the small number of confirmed cases investigated in this report. Taken together, these factors create a critical vulnerability for response to public health emergencies reminiscent of those encountered in the region during the 2018–2020 Ebola virus disease outbreak. While the DRC already faces extensive public health hurdles, including extreme economic and development hardships, these are exacerbated within IDP camps. The convergence of resource limitations within affected sites (including healthcare access, sanitation, clean water, food, and overcrowding) could facilitate rapid mpox circulation and potentially broaden this outbreak to a larger humanitarian crisis^32^. Consequently, the high risk of the mpox public health emergency to further expand nationally and internationally must be considered an urgent issue and a priority for all stakeholders. These cases involved adult males and females, as well as children, who were infected by MPXV through various transmission routes, including close non-sexual contacts. Our analysis demonstrates the continued expansion of Clade Ib MPXV and the first identification of cases within an IDP camp, highlighting the concerns for rapid expansion of the outbreak among highly vulnerable populations.

The spread of MPXV Clade Ib in the city of Goma, North Kivu, is highly concerning, as the virus has further expanded geographically within the DRC as well as more broadly, internationally. Beyond the nine investigated cases, the 170 suspected cases are also indicative of purported continued mpox spread in the region—with similar demographic and clinical presentations, these cases may signal increasing mpox risk in North Kivu province. This risk is exacerbated due to Goma’s proximity to porous international border regions, massive population displacement due to armed conflicts, and its international airport. The occurrence of MPXV Clade I in the societal background of eastern DRC requires improved countermeasures to quickly control the spread of the disease beyond internal and international borders^31–33^. Therefore, there is an urgent need for collaborative efforts and actions to control mpox before its further spread to other neighboring provinces and countries.

## Supplementary information

Description of Additional Supplementary Files

Supplemental Data 1

Reporting summary

Supplementary Table 1

## Data Availability

The source data for Figs. 3 and 4 can be accessed via the GenBank/ENA accession numbers provided in Supplementary Data 1. All other data are available from the corresponding authors upon reasonable request.
