# Supplementary material for: First imported cases of MPXV Clade Ib from Goma, Democratic Republic of the Congo
Source: Commun Med (Lond). 2025 Nov 15;5:496. doi: 10.1038/s43856-025-01203-z (PMC12647660; doi:10.1038/s43856-025-01203-z)
Supplement: Supplementary file 1 — Description of Additional Supplementary Files [file 43856_2025_1203_MOESM1_ESM.pdf]

Description of Additional Supplementary Files

File name: Supplementary data 1

Description: Clade Ib MPXV genomes represented in the phylogenetic tree in Figure 4.
